# Polio Surge Capacity Support Program Contributions to Building Country Capacities in Support of Polio Outbreak Preparedness and Response: Lessons Learned and Remaining Challenges

**DOI:** 10.3390/pathogens13050377

**Published:** 2024-05-01

**Authors:** Fikru Abebe, Victor Anochieboh Eboh, Mesfin Belew Weldetsadik, Ibrahima Kone, Tessema Assegid Kebede, Paul Thomas Harries, Veh Kesse Fabien Diomande

**Affiliations:** 1 Polio Surge Capacity Support Program, Center for Vaccine Equity (CVE), The Task Force for Global Health (TFGH), Decatur, GA 30030, USA; fabebe@taskforce.org (F.A.); veboh@taskforce.org (V.A.E.); mweldetsadik@taskforce.org (M.B.W.); kibrahima_consultant@taskforce.org (I.K.); tkebede-consultant@taskforce.org (T.A.K.); 2 Global Security Department (GSD), The Task Force for Global Health (TFGH), Decatur, GA 30030, USA; gsd@taskforce.org

**Keywords:** poliovirus, partnership, surveillance, campaign, vaccine, children

## Abstract

Despite coordinated efforts at global level, through the Global Polio Eradication Initiative (GPEI), poliomyelitis disease (Polio) is still a major public health issue. The wild poliovirus type-1 (WPV1) is still endemic in Afghanistan and Pakistan, and new circulations of the WPV1 were confirmed in southeast Africa in 2021, in Malawi and Mozambique. The circulating vaccine derived polioviruses (cVDPV) are also causing outbreaks worldwide. The Task Force for Global Health (TFGH)’s Polio Surge Capacity Support Program, established in 2019, is an effort to reinforce the existing partnership with the GPEI to strengthen countries’ capacities for polio outbreak preparedness and response. In four years, its coordinated efforts with GPEI partners have resulted in a remarkable improvement in the early detection of poliovirus circulation and reducing the missed children gaps in many countries. However, these encouraging results cannot hide an increasingly complex programmatic environment with numerous funding and operational challenges.

## 1. Introduction

The Global Polio Eradication Initiative (GPEI) has successfully reduced the poliomyelitis disease (Polio) burden globally by more than 99% worldwide [1,2]. The Polio Eradication End Game Strategic Plans (2013–2018 and 2019–2023) and the Polio Eradication Strategy 2022–2026 are aimed at eradicating Wild Poliovirus type-1 (WPV1) globally, as well as circulating Vaccine Derived Poliovirus type-2 (cVDPV2) simultaneously [3,4]. Only one serotype of wild poliovirus is still circulating and is endemic in Afghanistan and Pakistan [5]. In 2021, outbreaks of WPV1 were confirmed in southeast Africa (Malawi and Mozambique) [6,7]. In addition to WPV1, circulating Vaccine Derived Polioviruses (cVDPVs) continue to cause multiple outbreaks worldwide since 2000 [5,7]. The global poliovirus outbreak situation remains concerning despite efforts undertaken for the early detection of poliovirus circulation through strengthening country surveillance systems and reinforcing population immunity against the poliovirus through routine immunization services and supplemental immunization activities (SIA). In 2023, 1057 poliovirus positive samples were detected from Acute Flaccid Paralysis (AFP) cases and/or environmental samples worldwide (Africa, Asia, the Middle East, Europe, and America). Among them, 162 were WPV1 and 895 were circulating Vaccine Derived Poliovirus type-1 (cVDPV1) and cVDPV2 [8]. 

In 2019, the Task Force for Global Health (TFGH)’s Center for Vaccine Equity (CVE) and the US Centers for Disease Control and Prevention (CDC)’s Polio Eradication Branch (PEB) established the Polio Surge Capacity Support Program in partnership with the GPEI to develop and implement activities to strengthen the early detection and confirmation of poliovirus circulation, improve the performance and coverage of immunization services, reach under-immunized populations, strengthen the reach and quality of SIA, tailor strategies for high-risk areas and populations, and increase the demand for polio vaccination.

## 2. Support to Polio Outbreak Countries

The TFGH’s Polio Surge Capacity Support Program works closely with the US CDC and all GPEI partners at the country level through a collaborative platform to provide needed technical support to polio outbreak countries in support of their outbreak preparedness and response: country Ministry of Health, World Health Organization (WHO), United Nations International Children’s Emergency Fund (UNICEF), Bill and Melinda Gate Foundation (BMGF), and ROTARY. This includes a review of surveillance and immunization program data (SIA and routine immunization) to identify high-risk program areas, priority countries, and sub-national consequential geographies for surge support and capacity building. Technical support to polio outbreak countries follows GPEI Standard Operating Procedures (SOPs) [9] and provides training, monitoring of activities, and supervision and coaching of actors at all levels. Program areas targeted for support include:

- Coordination of the response at the country polio Emergency Operating Centers (EOCs)
- Strengthening of the surveillance systems
- Polio campaign preparation, implementation, and evaluation
- Outbreak response assessments, including defining the number of missed children and special populations (refugees, internally displaced populations, and populations living in security-compromised areas).

Over the past four years, the Polio Surge Capacity Support Program has performed 64 deployments of senior epidemiologists and local contractors in 16 polio outbreak countries in Africa, representing a cumulative 16 deployments per year and a total cumulative 167 months (almost 14 years) of fieldwork. Among the outbreak countries supported, five countries were classified as high-security risk at the time of deployments: Somalia, Ethiopia, Nigeria, South Sudan, and the Democratic Republic of Congo (DRC).

## 3. Security Risks and Mitigation Measures

Many polio outbreak countries being supported are at the highest security risk due to political unrest, terrorism, etc. Also, some countries with overall low or medium security risks could have pockets of high-risk areas at the sub-national level. Reaching all of the communities to bring them immunization and surveillance services and reaching all of the eligible children for polio vaccination, including those living in security-compromised areas, is a program priority. The deployments of Surge teams in high-security risk countries or security-compromised areas are performed through a detailed and careful security review and assessment by the TFGH Global Security Director (GSD). Security clearance for the deployment of the Surge team comes with security mitigation measures, which include the following activities and processes:

- All trips are entered into the Concur Travel system, and reports are generated for the TFGH Leadership and GSD.
- Travelers receive a guidance document including information on travel risks and reminders.
- The GSD assesses every trip, with higher-risk locations receiving increased scrutiny.
- Countries are risk assessed using Healix Sentinel (TFGH travel insurance provider), which uses a five (5) tier rating system (extreme, high, moderate, low, or minimal), and the US State Department Travel Advisory country risk rating system, which uses a four-tier system (Level: 4 Do Not Travel Level; 3 Reconsider Travel Level; 2 Exercise Increased Caution Level; and 1 Exercise Normal Precautions).
- Travelers are offered a pre-travel briefing before they travel.
- A three-day in-person Hostile Environment Awareness Training (HEAT) workshop is provided to the polio Surge staff.
- GSD has access to the Risk and Strategic Management Corps, within the SIGMA 7 (RSM/SIGMA 7) system provided by Intelligence Fusion, which provides a database of incidents recorded in different locations worldwide.
- GSD monitors TFGH staff using the Healix Sentinel mapping system.
- For higher-risk destinations, there is an additional layer where the traveler opens a WhatsApp Group, including the Program Leadership and GSD.
- GSD regularly contacts long-term deployed contractors and staff to discuss their safety and security.

## 4. Achievements 

Over the past four years, the Polio Surge Capacity Support Program, in close coordination with the US CDC PEB team and the GPEI partners, has assessed and prioritized technical support requests from polio outbreak countries and provided in-country human resource capacities where needed, with the recruitment and increased engagement of local contractors in Liberia, Senegal, Somalia, Ethiopia, Kenya, Malawi, Tanzania, and Zambia. Where needed, the program has also reinforced in-country human resource capacity with the deployments of senior epidemiologists in Ghana, Sierra Leone, Senegal, Gambia, Somalia, Djibouti, Ethiopia, Kenya, Tanzania, Zambia, Malawi, DRC, and Nigeria. 

**Figure 1:**
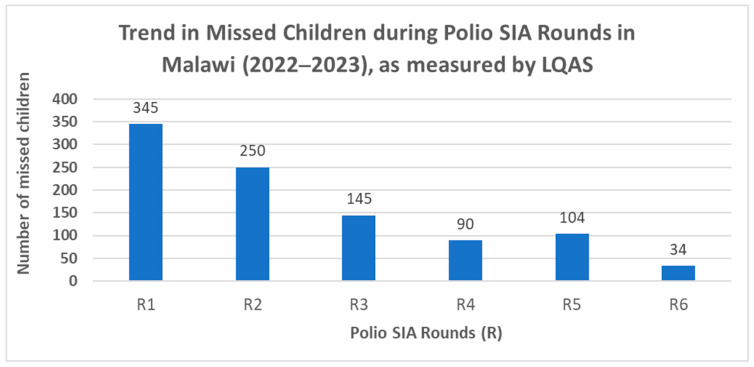
Trends in missed children during Polio SIA in Malawi (2022–2023), as measured by LQAS. Notes: SIA—supplementary immunization activities; LQAS—lot quality assurance sampling.

The collaborative efforts between the Polio Surge Capacity Support Program and the US CDC PEB, in close coordination with GPEI partners, have enabled improvement of the early detection of poliovirus circulation in many countries, and contributed to the outbreak countries’ efforts to reach and vaccinate cumulatively over two hundred million children against polio over the past four years in sixteen countries in Africa. These remarkable results are illustrated by the trend in the reduction in missed children for polio vaccination in SIAs during the Southeast Africa outbreak response from 2022 to 2023 in Malawi (Figure 1). Similar trends in progressively reducing the number of missed children have also been observed in several supported outbreak countries, such as Djibouti, Somalia, Tanzania, Zambia, and the DRC. 

## 5. Growing Program Complexity with Persistent Challenges

Program complexity has grown and is related to several internal and external factors that negatively impact the program’s effectiveness at reaching all the communities and eligible children for polio vaccination and surveillance activities. The major persistent challenges are the following:

- The lack of sufficient funding to address countries’ growing technical assistance needs is a result of insufficient government contributions to polio outbreak response and the reduction of the funding stream at a global level. To address funding gaps, the Polio Surge Capacity Support Program regularly conducts prioritization exercises in close coordination with the CDC and other GPEI partners to determine a list of high-priority countries to be supported for outbreak preparedness and response: countries with a circulation of WPV. These “wide-spreader” countries are defined as countries with factors heightening the risk of widespread of poliovirus (poor disease surveillance leading to undetected transmission, growing vaccine hesitancy, large proportion of unvaccinated populations, important population movements, etc.) and countries with no recent experience with polio outbreak responses. The program resources are then redirected to the highest priority countries, leaving other priority countries without support to sustain previous gains against polio (the early detection of poliovirus circulation and sustaining population immunity against poliovirus). The lack of sufficient funding to address countries’ needs for outbreak preparedness and response represents a serious threat to polio eradication efforts.
- The observed limited vaccine supplies of the novel Oral Polio Vaccine type-2 (nOPV2) [10], as documented in many country outbreak response situation reports (SitReps), is impacting polio campaign schedules, resulting in a delay in the implementation and a reduction in the rounds of the campaigns. The lack of sufficient vaccines to address country needs represents a major challenge in increasing population immunity to prevent continuing the transmission of VDPV2.
- Inadequate country-level engagement: The country-level engagement for polio outbreak response by national authorities at all levels is still inadequate in most outbreak countries, mainly related to competing public priorities and other post-COVID-19 pandemic socio-economic priorities. This leads to poor ownership, the absence of an accountability framework, a lack of supportive supervision during SIAs and surveillance activities, and late disbursement of resources at operational levels. Poor engagement and lack of ownership represent a major challenge in reaching all communities and eligible children for polio vaccination.
- The lack of adequate funding and poor engagement of national authorities for polio outbreak response contribute to persistent operational constraints and inadequate program performance. This results in late detection and laboratory confirmation of poliovirus circulations, late deployments of human resources to outbreak countries, late disbursement of funds to operational levels, delays in service delivery, lack of adequate microplanning, weak cross-border collaborations, and lack of effective measures to track and address persistent rumors against vaccination and/or vaccine hesitancies.
- The underlying growing burden of epidemic diseases in developing countries, the post-COVID-19 socio-economic impacts, and the impact of emerging military crises worldwide on the global economy are major factors influencing leadership decision-making not to prioritize disease outbreak preparedness and response, including polio.
- As described above, the need for adequate resources to simultaneously address the growing public health and development needs is a major challenge for disease epidemic preparedness and response. This context of competing public health emergencies and priorities is fueling weak country-level engagement and ownership and poor government contributions to the funding of polio outbreak response. Most of the polio outbreak countries are still facing multiple public health emergencies at the same time (measles, cholera, viral hemorrhagic fever outbreaks, natural disasters, etc.). Inaccurate population figures, due to the lack of reliable census data, worsen this situation and affect the quality of the planning of activities and the accuracy of coverage results.
- Growing security risks in many countries has resulted in political instability, leading to poor engagement and commitment of local authorities. The resulting conflict exacerbates access issues due to population movements (refugees, internally displaced populations (IDPs)), wide geographies and difficult terrain resulting in chronically missed children, poor vaccination coverage, continued circulation of poliovirus, and spreading of outbreaks.
- Chronically missed children for vaccination: The interactions between the multiple factors above described lead to the late detection of poliovirus circulation, resulting in the late and poor planning and implementation of polio outbreak preparedness and responses in many countries. The persistent poor SIA quality in many outbreak settings and the existence of many pockets of underserved populations (insecurity, difficult terrains, displaced populations, and populations with persistent rumors against vaccination and/or vaccine hesitancies) are the main contributing factors to the chronically missed children for vaccination that is observed.

## 6. Conclusions

The Polio Surge Capacity Support Program collaborative platform with the US CDC and GPEI partners for polio outbreak preparedness and response is providing a unique opportunity for the routine review of country technical assistance needs, program performance review, and ability to provide technical assistance needs in the context of available resources. This collaborative platform continues to effectively support polio outbreak countries in improving the early detection of poliovirus circulation and narrowing the missed children’s gaps. The above-described growing program complexity and challenges require increased resources and strong measures to allow for the effective eradication of polio outbreaks. Among these measures, priority should be provided to strongly advocating GPEI partners and member states in order to continue to mobilize more resources for polio eradication, especially to strengthen the mobilization of local resources by member states to complement GPEI efforts. Urgent attention should also be provided to review the identified emerging program challenges, as described above, and to determine realistic and country-specific action plans to address them. 

## Data Availability

Restrictions apply to the availability of these data. Data were obtained from the Situation Reports of outbreak countries, and are available from the authors with the permission of countries.
